# A Case of Hypoxemic Primary Cytomegalovirus Disease Shortly After Recovery From Coronavirus Disease in a Young Immunocompetent Man

**DOI:** 10.1155/crdi/7915155

**Published:** 2025-10-09

**Authors:** Yoshihiro Kitahara, Kanako Nakamoto, Yusuke Takayama, Kei Miwata, Goki Ushio, Shoshi Akieda, Toshiro Takafuta

**Affiliations:** Department of Internal Medicine, Hiroshima City Funairi Citizens Hospital, 14-11 Funairi-saiwai-cho, Naka-ku, Hiroshima 730-0844, Hiroshima, Japan

**Keywords:** CMV antigenemia, CMV IgG, CMV IgM, coronavirus disease, cytomegalovirus, deoxyribonucleic acid of CMV, primary CMV disease, young immunocompetent patients with COVID-19

## Abstract

Cytomegalovirus (CMV) reactivation has been identified as a significant predictor of death in patients hospitalized with coronavirus disease (COVID-19). However, reports of concurrent or subsequent primary CMV disease in young immunocompetent patients with COVID-19 are rare. We present a rare case of primary CMV disease manifesting as pneumonia and acute respiratory failure in a young immunocompetent man, which developed shortly after recovery from moderate COVID-19. A 19-year-old immunocompetent man was admitted to our hospital with a diagnosis of COVID-19 pneumonia on the 10^th^ day of COVID-19 onset. His symptoms improved following administration of intravenous dexamethasone, oral prednisolone, and oral azithromycin hydrate and levofloxacin hydrate. He was discharged on the 16^th^ day of COVID-19 onset. However, he developed a cough and fever 5 days after discharge, and he was readmitted to our hospital 8 days after discharge. Serum levels of aspartate aminotransferase (AST), alanine aminotransferase (ALT), C-reactive protein (CRP), and lactate dehydrogenase (LDH) were elevated compared to those during the first admission. Computed tomography revealed new ground-glass attenuations and the improvement in the aforementioned COVID-19 pneumonia. Next day, his percutaneous arterial oxygen saturation levels dropped to 89% breathing room air. Serum antibodies against CMV: immunoglobulin M (CMV IgM) and immunoglobulin G (CMV IgG), which were negative on the day of the first admission, became positive (CMV IgM titer, 5.82 [sample relative light units/cutoff] and CMV IgG titer, 58.6 [arbitrary units/mL]) on the 3^rd^ day of the second admission. The patient was diagnosed with primary CMV disease based on positive test results of the CMV antigenemia and deoxyribonucleic acid of CMV. The patient's symptoms, hypoxemia, and the new ground-glass attenuations improved following intravenous ganciclovir administration, without using corticosteroids. The clinical course in the present case suggests that CMV could have been the dominant causative pathogen for the new pneumonic shadows observed on the second admission. In cases where a second fever occurs shortly after recovery from COVID-19, clinicians should not assume prolonged COVID-19, and primary CMV disease should be considered as a differential diagnosis, even in young immunocompetent patients.

## 1. Introduction

Cytomegalovirus (CMV), also known as human herpesvirus 5, is the largest human herpesvirus [1, 2]. Most individuals are infected with CMV during childhood via body fluids such as blood, breast milk, urine, and saliva. Patients typically become positive for serum antibodies against CMV: immunoglobulin M (CMV IgM) and immunoglobulin G (CMV IgG), approximately 1-2 and 2-3 weeks after primary CMV infection, respectively [3]. Approximately 60% of adults in developed countries and more than 90% of adults in developing countries are positive for CMV IgG [1, 2]. After the primary infection, CMV remains latent in granulocyte-macrophage progenitor cells derived from the bone marrow or embryonic liver [4]. Although most primary CMV infections in young immunocompetent individuals are asymptomatic, some cases progress to primary CMV disease, presenting with symptoms that resemble those of infectious mononucleosis [5], or symptoms by inflammation involving various organs such as the gastrointestinal tract, central nervous system, ocular tissue, and lung [6].

Prior to the approval of ganciclovir in 1988, CMV disease could be fatal even in immunocompetent patients. However, it is now treatable using ganciclovir or valganciclovir hydrochloride [7]. Nevertheless, CMV pneumonia can still be fatal as an opportunistic infectious disease via CMV reactivation in immunocompromised patients, including older patients, those with malignancies or acquired immunodeficiency syndrome, or those undergoing immunosuppressive treatment [8]. Pérez-Granda et al. [9] reported CMV reactivation at the time of hospital admission as a significant predictor of mortality in patients with coronavirus disease (COVID-19) with pneumonia and hypoxemia. Their study, which included patients with a relatively high median age of 64.00 (53.25–76.00) years, suggested a reciprocal effect between COVID-19 and CMV reactivation in older patients: COVID-19 may weaken immunity, leading to CMV reactivation and subsequent pneumonia, which results in a poor prognosis in this population.

In contrast, reports on concurrent or subsequent primary CMV disease in young immunocompetent patients with COVID-19 are limited. Recently, Ura et al. [10] reported the co-occurrence of CMV disease in a 36-year-old man with mild COVID-19 who had a persistent fever lasting 4 weeks. The authors speculated that immune dysfunction had occurred owing to uncontrolled diabetes mellitus with a high glycosylated hemoglobin level of 10.9% [10]. They suspected primary CMV infection based on the time course of changes in CMV IgM and IgG titers but could not rule out CMV reactivation strictly because the patient was positive for CMV IgG from the first measurement, with a titer of 8.5 arbitrary units (AU)/mL [10].

On May 8, 2023, the classification of COVID-19 was changed from Class 2 to Class 5 under the Infectious Diseases Control Law in Japan [11]. Despite this change, the number of patients with COVID-19 has continued to increase in periodic waves [12]. Considering that the prevalence of CMV antibodies in Japanese adults was 80% to 90% in the 1970s–1980s and decreased to 70% in 2008-2009 [13], primary CMV disease can occur concurrently with COVID-19 or subsequently to COVID-19 in young immunocompetent subjects, depending on the timing of the infection with CMV and severe acute respiratory syndrome coronavirus 2 (SARS-CoV-2).

Herein, we report a rare case of primary CMV disease in a young immunocompetent man that occurred shortly after his recovery from COVID-19. By means of repeated measurements using the leftover serum, we observed the conversion to CMV IgG positivity and a gradual increase in the CMV IgM titer. This confirmed that the CMV disease developed due to a primary CMV infection rather than CMV reactivation, and we presumed that the patient was still in the incubation period for primary CMV disease at the time of the COVID-19 onset.

## 2. Case Presentation

A 19-year-old man started experiencing pharyngeal discomfort during mid-January 2021. He was diagnosed with COVID-19 based on a positive polymerase chain reaction (PCR) test for SARS-CoV-2 with a cycle threshold value of 33 on the 4^th^ day of disease onset (with the day of the COVID-19 onset counted as the “0^th^ day”) (Table 1). While isolated in a hotel, following the policy of the Japanese Ministry of Health, Labour and Welfare, he developed a cough and high fever, and was admitted to our hospital on the 10^th^ day of the COVID-19 onset. He was a never-smoker with no medical history of note. He had never experienced sexual intercourse, and had no medical history suggestive of an immunodeficiency syndrome.

**Figure 1:**
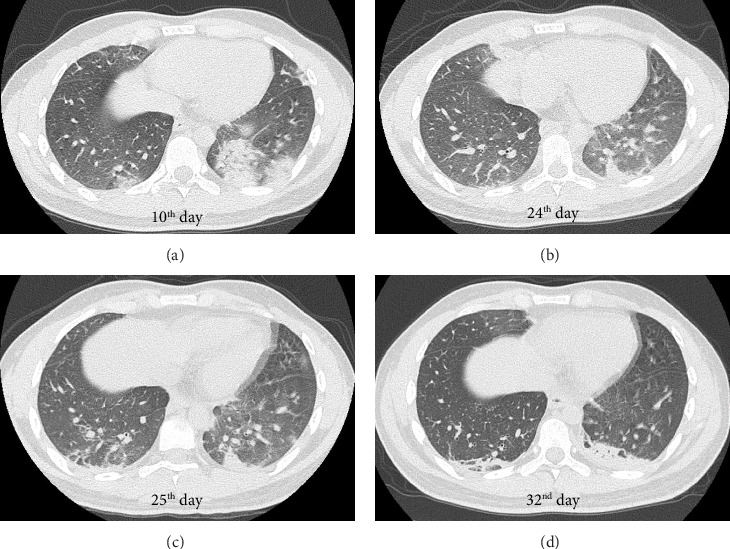
Changes in the chest computed tomography (CT) images. ^∗^The scanning level is different between (a) and (b–d). The level of scanning in (a) is more cranial compared to that in (b–d). ^#^Note that the day of coronavirus disease (COVID-19) onset is counted as the “0^th^ day.” (a) CT image on the day of the first admission (10^th^ day of the COVID-19 onset), showing multiple infiltrative shadows in both lung fields, suggestive of COVID-19 pneumonia with or without secondary bacterial pneumonia. (b) CT image on the day of the second admission (24^th^ day of the COVID-19 onset), showing improvement in bilateral infiltrative shadows. A crescent moon-shaped consolidation remained in the left-lower lung lobe, and ground-glass attenuations newly appeared in both lower lung lobes. (c) CT image on the 2^nd^ day of the second admission (25^th^ day of the COVID-19 onset), showing new linear consolidations on the dorsal side of the right lower lung lobe. (d) CT image on the 9^th^ day of the second admission (32^nd^ day of the COVID-19 onset). Ground-glass attenuations improved, and linear consolidations spread to a plate shape in both lower lung lobes, which was suggestive of organizing changes.

On admission, the patient exhibited a clear level of consciousness. His body mass index was 21.1 kg/m^2^. His vital signs included a body temperature (BT) of 38.0°C, a blood pressure of 120/71 mmHg, a pulse rate of 118 beats/min with a regular rhythm, and a slight decrease in the percutaneous arterial oxygen saturation (SpO_2_) of 96% breathing room air. Although he complained of slight dyspnea, his respiratory sounds were normal, without crackles or wheezing. His heart sounds were also normal without murmurs. No peripheral skin lesions, lymph node swelling, or edema were observed. Computed tomography (CT) revealed multiple infiltrative shadows in both lung fields suggestive of COVID-19 pneumonia with or without secondary bacterial pneumonia (Figure 1(a)). The severity of COVID-19 was defined as moderate I [14].

**Figure 2:**
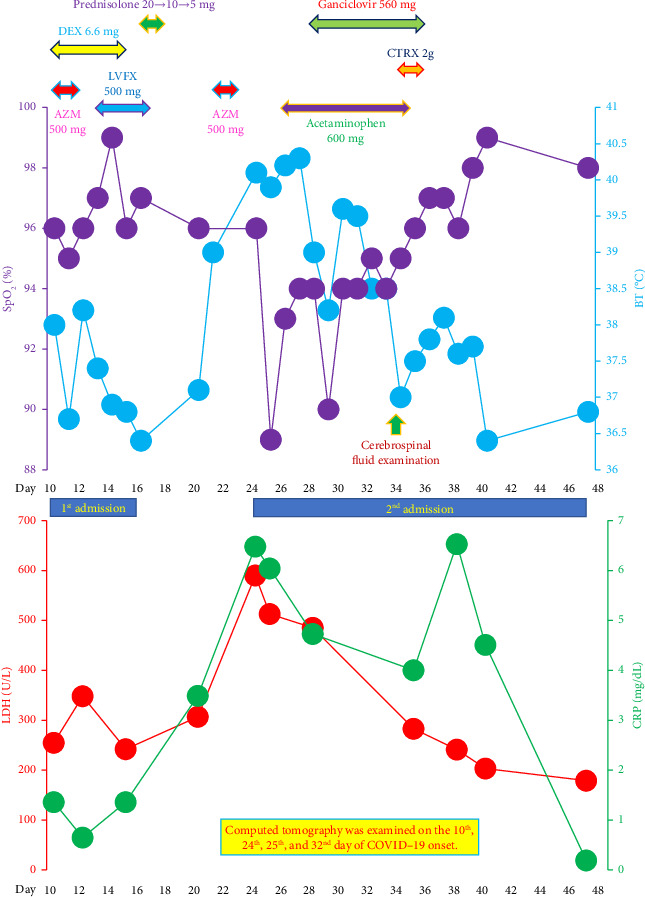
Course in the patient body temperature (BT), percutaneous arterial oxygen saturation (SpO_2_), serum lactate dehydrogenase (LDH) levels, and serum C-reactive protein (CRP) levels over time. ^∗^Note that “Day” in the figure represents the number of the day from coronavirus disease (COVID-19) onset. The day of COVID-19 onset is counted as the “0^th^ day.” The duration of the first admission was 5 days (from the 10^th^ to the 16^th^ day of the COVID-19 onset). Medication during the first admission was as follows: intravenous administration of dexamethasone (DEX) 6.6 mg/day (from the 10^th^ to the 15^th^ day), oral prednisolone (20 mg on the 16^th^ day, 10 mg on the 17^th^ day, 5 mg on the 18^th^ day), oral azithromycin hydrate (AZM) 500 mg/day (from the 10^th^ to the 12^th^ day), and oral levofloxacin hydrate (LVFX) 500 mg/day (from the 13^th^ to the 17^th^ day). Although fever and SpO_2_ improved with the medication, serum levels of CRP (1.36 mg/dL) and LDH (255 U/L) were almost the same on discharge (CRP, 1.36 mg/dL and LDH, 242 U/L). The duration of the outpatient care between the first and second admissions was 7 days (from the 17^th^ to the 23^rd^ day of the COVID-19 onset). Medication in this period was as follows: oral AZM 500 mg/day (from the 21^st^ to the 23^rd^ day). The duration of the second admission was 24 days (from the 24^th^ to the 47^th^ day of the COVID-19 onset). Medication during the second admission was as follows: oral acetaminophen 600 mg/day (from the 26^th^ to the 35^th^ day), intravenous ganciclovir 560 mg/day (from the 28^th^ to the 36^th^ day), and ceftriaxone sodium hydrate (CTRX) 2 g/day (from the 34^th^ to the 36^th^ day). Cerebrospinal fluid examination was performed on the 34^th^ day of the COVID-19 onset. On the day of the second admission, serum levels of CRP (6.48 mg/dL) and LDH (590 U/L) were considerably higher than those recorded on the day of the first admission. During the second admission, the highest BT was 40.3°C, and the lowest SpO_2_ was 89% breathing room air. Following intravenous administration of ganciclovir, the serum level of CRP decreased to 0.19 mg/dL and that of LDH normalized to 179 U/L, with improvement in fever and SpO_2_.

Laboratory tests showed a normal white blood cell count (4900 cells/μL) with a normal percentage of neutrophils (53.5%) and lymphocytes (34.0%) and increased atypical lymphocytes (3.5%). Although serum levels of aspartate aminotransferase (AST, 25 U/L), alanine aminotransferase (ALT, 24 U/L), and γ-glutamyl transpeptidase (γ-GTP, 44 U/L) were within normal limits, those of serum C-reactive protein (CRP, 1.36 mg/dL), lactate dehydrogenase (LDH, 255 U/L), and soluble interleukin-2 receptor (sIL-2R, 650 U/mL) were mildly elevated (Figure 2). Qualitative tests for surface antigen, surface antibody, and core antibody of hepatitis B virus were negative. The antibody titer (majority IgM) against *Mycoplasma pneumoniae*, determined using the particle agglutination method, were ×80, below the minimum level (×320) required for a definitive diagnosis of infection with this microorganism in a single measurement.

Oral azithromycin hydrate (AZM, 500 mg/day, once a day for 3 days) and levofloxacin hydrate (LVFX, 500 mg/day, once a day for 5 days) were administered sequentially to treat latent secondary bacterial infections (Figure 2). Although the decrease in SpO_2_ was slight, with the lowest value of 95%, the intravenous administration of dexamethasone (DEX, 6.6 mg/day) was initiated on the day of admission (10^th^ day of the COVID-19 onset) to treat his prolonged fever and cough (Figure 2) [14]. On the 14^th^ day of the COVID-19 onset, his BT declined to below 37.0°C without antipyretics and his cough improved. His SpO_2_ increased to 97%–99% breathing room air.

Serum levels of CRP (1.36 mg/dL) and LDH (242 U/L) were almost equal to those on admission (Figure 2). The patient was discharged on the 16^th^ day of the COVID-19 onset (7^th^ day of admission). Intravenous DEX was continued for 6 days (until the 15^th^ day of the COVID-19 onset), and then the dose of corticosteroids was tapered for 3 days with oral prednisolone (20, 10, and 5 mg/day, respectively). Thus, corticosteroids therapy was continued for 9 days until 2 days after discharge (18^th^ day of the COVID-19 onset) (Figure 2).

Five days after discharge (21^st^ day of the COVID-19 onset), the patient developed a dry cough and a high fever of 39.0°C. The fever persisted despite administration of acetaminophen as an antipyretic and represcription of AZM (from the 21^st^ to the 23^rd^ day of the COVID-19 onset), and he was readmitted to our hospital 3 days later (24^th^ day of the COVID-19 onset). On the day of the second admission, the patient had a high BT of 40.1°C, a blood pressure of 105/64 mmHg, and a pulse rate of 140 beats/min with a regular rhythm. The patient did not show hypoxemia, with an SpO_2_ of 96% breathing room air. His neck and submandibular lymph nodes were swollen.

A follow-up CT examination on the day of the second admission revealed improvement in the bilateral infiltrative shadows with a crescent moon-shaped consolidation persisting in the left lower lung lobe (Figure 1(b)). Ground-glass attenuations newly appeared in both lower lung lobes (Figure 1(b)). Laboratory tests showed an increased white blood cell count (11,100 cells/μL) with a normal neutrophil percentage (46.7%) and a low eosinophil percentage (0.3%). Although the lymphocyte percentage increased to 48.1%, it remained within the normal range (18%–53%). A slightly elevated percentage of atypical lymphocytes (4.5%) was also noted. Along with the elevation in the serum levels of AST (137 U/L), ALT (273 U/L), and γ-GTP (148 U/L), the serum levels of CRP (6.48 mg/dL), LDH (590 U/L), and sIL-2R (1683 U/mL) were elevated compared to those on the day of the first admission. The *Mycoplasma pneumoniae* antibody titer remained unchanged (×80). The titer of IgM against *Chlamydophila pneumoniae*, determined using enzyme-linked immunosorbent assay, was not elevated (0.1, normal range: 0.0–0.5).

His cough improved with the intravenous administration of hydroxyzine hydrochloride and theophylline. However, his BT remained high (39.9°C) on the next day (25^th^ day of the COVID-19 onset), which was accompanied by a headache and hypoxemia, with an SpO_2_ of 89% breathing room air. The PCR test result for SARS-CoV-2 detection using a nasopharyngeal swab was negative. We performed CT examination again and found new linear consolidations on the dorsal side of the right lower lung lobe (Figure 1(c)). Based on the CT findings, we considered it reasonable to suspect recurrent COVID-19 pneumonia and secondary bacterial pneumonia. However, the higher serum levels of AST, ALT, and LDH and higher percentage of lymphocytes and atypical lymphocytes on the second admission compared to that on the first admission led us to suspect other viral coinfections.

On the 24^th^ day of the COVID-19 onset, CMV antigenemia testing, which detects CMV pp65 antigen-positive polymorphonuclear leukocytes [15], was positive (positive white blood cell counts: 8–13/50,000 white blood cells) (Table 1). On the 26^th^ day of the COVID-19 onset, a quantitative PCR test for deoxyribonucleic acid of CMV (CMV DNA) was performed using a TaqMan PCR assay [15], which was also positive (3.9 logarithm international unit [Log IU]/mL, normal range: 0–1.49 Log IU/mL) (Table 1). CMV IgM and IgG titers were measured via fully automated chemiluminescence immunoassay: ARCHITECT® CMV-M・Abbott kit and ARCHITECT® CMV-G・Abbott kit, measured with an ARCHITECT® analyzer i2000SR (Abbott Japan LLC., Tokyo, Japan). The patient was positive for both CMV IgM and CMV IgG on the 26^th^ day of the COVID-19 onset, with the following antibody titers: CMV IgM, 5.82 sample relative light units/cutoff [S/CO] (normal range: 0.00–0.85 S/CO) and CMV IgG, 58.6 AU/mL (normal range: 0.0–6.0 AU/mL) (Table 1). To determine whether the condition of the patient was the result of a primary CMV infection or the reactivation of CMV, we measured CMV IgM and CMV IgG using the leftover serum samples collected on the day of the first admission (10^th^ day of the COVID-19 onset) and found that both CMV IgM and CMV IgG were negative with the following antibody titers: CMV IgM, 0.35 S/CO and CMV IgG, < 6.0 AU/mL (Table 1). These findings confirmed primary CMV infection as a causative etiology for the second fever episode from the 21^st^ day of the COVID-19 onset (Figure 2) [5].

Fluorescent antibody technique was used to detect antibodies against Epstein–Barr virus (EBV). The IgM and IgG titers against the EBV viral capsid antigen were < ×10 (normal range: ×0 − 10) and ×320 (normal range: ×0 − 10), respectively. The IgG titers against EBV nuclear antigen was ×40 (normal range: ×0 − 10). These results suggested that he had been infected with EBV in the past and we could rule out a current EBV infection.

Although we speculated that CMV might have been an additional causative pathogen for the pneumonia, he refused our proposal to perform fiberoptic bronchoscopy to detect CMV DNA in bronchial lavage fluid or intranuclear inclusion bodies in the transbronchial lung biopsy specimen. We initiated an intravenous ganciclovir administration (280 mg per dose, twice a day) for 9 days, from the 28^th^ to the 36^th^ day of the COVID-19 onset (Figure 2) [8]. We prescribed acetaminophen (200 mg per dose, three times a day) from the 26^th^ to the 35^th^ day of the COVID-19 onset as an antipyretic to treat the persistent fever (Figure 2).

Although his fever improved on the 7^th^ day of the ganciclovir treatment (34^th^ day of the COVID-19 onset), his headache persisted, leading us to suspect CMV meningitis. On the 34^th^ day of the COVID-19 onset, we performed a cerebrospinal fluid (CSF) examination and initiated intravenous administration of ceftriaxone sodium hydrate (CTRX, 2 g per dose, once a day) for latent bacterial meningitis (Figure 2). The CSF was colorless and transparent. Based on the fact that LDH levels (14 U/L, normal range: 5–20 U/L), total protein levels (26.8 mg/dL, normal range: 10–40 mg/dL), and glucose levels (63 mg/dL, normal range: 40–75 mg/dL) in CSF were normal, and that neither mononuclear nor polymorphonuclear cell counts (3 and 0, respectively) increased in CSF, we ruled out the possibility of CMV meningitis. CTRX was administered from the 34^th^ to the 36^th^ day of the COVID-19 onset (Figure 2). Although his fever reappeared after CSF examination, it improved afterward and his headache gradually subsided (Figure 2).

A CT examination on the 32^nd^ day of the COVID-19 onset revealed that ground-glass attenuations improved, and linear consolidations spread to a plate shape in both lower lung lobes, which was suggestive of organizing changes (Figure 1(d)).

Serum levels of AST, ALT, and γ-GTP gradually decreased. On the 47^th^ day of the COVID-19 onset, the serum level of CRP decreased to 0.19 mg/dL and that of LDH normalized to 179 U/L. On the same day, the CMV IgM titer mildly decreased to 4.69 S/CO, and the CMV IgG titer increased to 110 AU/mL (Table 1). The positive white blood cell count in the CMV antigenemia test decreased to 0–1/50,000 white blood cells on the 32^nd^ and the 38^th^ days of the COVID-19 onset (Table 1). The screening tests for human immunodeficiency virus (HIV) measuring HIV-1 antibodies, HIV-2 antibodies, and HIV p24 antigen were negative. No widespread infection among his family or friends was observed.

## 3. Discussion

The most important clinical point in the present case was whether we should have suspected a CMV disease when the second fever appeared. Based on the improvements in existing pneumonic shadows and the appearance of new shadows, clinicians might suspect a combination of recurrent COVID-19 pneumonia and secondary bacterial pneumonia.

If the patient had not previously been diagnosed with COVID-19, it might have been easier to suspect a primary CMV disease in a young adult exhibiting fever, with an increase in atypical lymphocyte and transaminase levels [16]. Although these laboratory abnormalities can also occur in patients with COVID-19 [17, 18], we found it unusual that atypical lymphocytes and transaminase levels remained high even after the PCR test results for SARS-CoV-2 became negative.

In the present case, CMV and EBV antibody examinations were performed to rule out the involvement of other viral infections, and it was not until we obtained a positive result for CMV IgM that we suspected a CMV disease. However, it must be noted that the false-positive rate of CMV IgM seropositivity has been reported to range from 20.4% to 40.9% [19, 20], and that CMV IgM seropositivity is present in approximately one-third of primary EBV infections because of a one-way cross-reactivity [21]. In the present case, we discarded the possibility that CMV IgM seropositivity was not false-positive based on the positive result for CMV DNA and CMV antigenemia, and the negative result for antibodies against EBV.

Determining whether the CMV disease developed owing to a primary CMV infection or CMV reactivation is important. In the report by Pérez-Granda et al. [9], the diagnosis of CMV reactivation was based on the speculation of a previous CMV infection because the patients with COVID-19 and positive result for CMV DNA or CMV antigenemia were relatively older in age as described above. However, evaluation of the time-course changes in the CMV IgM and IgG titers is necessary to strictly differentiate between a primary infection and reactivation. In the present case, the availability of the leftover serum samples enabled us to perform this evaluation. We diagnosed a primary CMV disease and ruled out the possibility of CMV reactivation based on the negative result for CMV IgG on the first admission, a gradual increase in CMV IgM titer during the first admission, and the positive result for CMV IgG on the second admission.

The next issue to consider is the timeline of CMV and SARS-CoV-2 infections. In the present case, the infection pathway remained unclear. The incubation period for the perinatal CMV disease is 3–12 weeks after delivery, and in adult iatrogenic infections, CMV disease typically manifests 3–8 weeks after blood transfusion and 4–16 weeks after organ transplantation [22]. Although determining the incubation period in sporadic cases of primary CMV infection is difficult, it has been estimated to be approximately 4–6 weeks [19], which is considerably longer than the incubation period of 4–6 days for COVID-19 [23].

Assuming that this estimation is correct, and considering that the second episode of fever occurred approximately 3 weeks after the COVID-19 onset, the CMV infection was likely to have been established 1–3 weeks prior to the COVID-19 onset, and the patient was presumably in the incubation period for the primary CMV disease at the time of the COVID-19 onset. Additionally, given that he was still negative for CMV IgM on the 10^th^ day of the COVID-19 onset, the CMV infection was probably established between 1 week prior to the COVID-19 onset and a few days prior to the SARS-CoV-2 infection. In contrast, assuming that the incubation period in sporadic cases of a primary CMV infection might be shorter than the estimation described above, it is possible that the patient could have been infected with the two viruses concurrently, or that he could have been infected with CMV after SARS-CoV-2 infection or COVID-19 onset. However, any of these possibilities as for the timeline of CMV infection, SARS-CoV-2 infection, and COVID-19 onset remains speculative in the present case. Further studies with the accumulation of primary CMV disease cases are warranted to clarify the incubation period in sporadic cases of primary CMV infection.

A study of CMV pneumonia by Franquet et al. [24] revealed that the most frequent CT findings were bilateral ground-glass attenuations, with consolidations and nodular shadows also being typical, and that the lower lung lobes were the most common locations. Although we were unable to confirm the presence of CMV in lung tissues, the new ground-glass attenuations on CT at the onset of primary CMV disease were compatible with a diagnosis of CMV pneumonia (Figure 1(b)). Moreover, the ground-glass attenuations and hypoxemia improved after the administration of ganciclovir without using corticosteroids, a key drug for the treatment of hypoxemic COVID-19 [14]. The serum LDH level, which was higher on the second admission compared to that on the first admission, is a poor prognostic factor in patients with COVID-19 [25]. If recurrent COVID-19 pneumonia was a major component of the pneumonic shadows observed on the second admission, it is unreasonable that they could have improved without corticosteroids administration (Figures 1(d), 2). Therefore, the clinical course in the present case suggests that CMV could have been the dominant causative pathogen for these new pneumonic shadows observed on the second admission.

Franquet et al. [24] also reported that ground-glass attenuations or consolidations on CT correspond to the areas of exudative or proliferative phases of diffuse alveolar damage in pathological findings. However, we speculated that the decrease in ground-glass attenuations and replacement of consolidations after ganciclovir administration might reflect organizing changes in CMV pneumonia [26].

Japanese COVID-19 guidelines recommend using corticosteroids only in case the severity of COVID-19 is relevant to moderate II (SpO_2_ < 93%) or severe (requiring mechanical ventilation) [14]. Although the patient desaturation was relatively slight and did not meet the criteria for corticosteroids treatment [14], we administered corticosteroids for 9 days to treat prolonged fever and cough due to COVID-19 and observed an improvement in these symptoms. In contrast, the use of corticosteroids can cause CMV reactivation [27]. Amiya et al. [28] reported a fatal case of CMV pneumonia in an 80‐year‐old woman shortly after treatment for severe COVID-19 with corticosteroids and mechanical ventilation. While the patient in the present case was a young immunocompetent man, we considered the possibility that corticosteroids treatment might have increased the likelihood of a primary CMV disease onset by weakening his immune system [29].

In addition, this case report has clinical significance for similar cases occurring in pregnant women. If preceding COVID-19 is considered as an etiology of the second fever episode without the recognition of a primary CMV infection, there is a high risk of congenital CMV infection in newborns [30].

The coinfection rate with other viruses in patients with COVID-19 is approximately 3%, with respiratory syncytial virus and influenza virus A being the most prevalent, each accounting for approximately 15% of coinfections [31]. In contrast, coinfection with CMV in patients with COVID-19 is rare and accounts for approximately 1% of coinfections [31]. Recently, a multiplex-nested PCR test using a nasopharyngeal swab was developed, which enables the detection of SARS-CoV-2 and multiple viral coinfections within 1 h [32]. However, as CMV is not included as a target pathogen in this test kit, incidental detection of a CMV infection cannot be expected [32]. In cases in which a second fever episode occurs shortly after recovery from COVID-19, clinicians should not assume prolonged COVID-19, and primary CMV disease should be considered as a differential diagnosis, even in young immunocompetent patients.

## Figures and Tables

**Table 1:** Course in the test results for CMV and SARS-CoV-2 infection over time.

	**Number of the day from COVID-19 onset** ^**#**^					
	**10^th^**	**24^th^**	**26^th^**	**32^nd^**	**38^th^**	**47^th^**
CMV IgM (S/CO)	0.35^∗^		5.82			4.69
CMV IgG (AU/mL)	< 6.0^∗^		58.6			110
CMV antigenemia		+		+	+	
Positive cell Count 1 (/50,000 white blood cells)		13		0	0	
Positive cell Count 2 (/50,000 white blood cells)		8		1	1	
CMV DNA (Log IU/mL)			3.9			
PCR test for SARS-CoV-2	+	−				
Cycle threshold value of PCR test for SARS-CoV-2	33.3					

*Note:* CMV: cytomegalovirus; COVID-19: coronavirus disease, S/CO: sample relative light units/cutoff.

Abbreviations: AU/mL, arbitrary units/milliliter; DNA, deoxyribonucleic acid; IgG, immunoglobulin G; IgM, immunoglobulin M; Log IU/mL, logarithm international unit/milliliter; PCR, polymerase chain reaction; SARS-CoV-2, severe acute respiratory syndrome coronavirus 2.

^#^The day of COVID-19 onset is counted as the “0^th^ day.”

^∗^CMV IgM and CMV IgG titers on the 10^th^ day of COVID-19 onset were measured using leftover serum.

## Data Availability

The data that support the findings of this study are openly available in [repository name] at [DOI].
